# Transcriptional landscape of human cancers

**DOI:** 10.18632/oncotarget.15837

**Published:** 2017-03-02

**Authors:** Mengyuan Li, Qingrong Sun, Xiaosheng Wang

**Affiliations:** ^1^ School of Science, China Pharmaceutical University, Nanjing 211198, China; ^2^ Department of Basic Medicine, School of Basic Medicine and Clinical Pharmacy, China Pharmaceutical University, Nanjing 211198, China

**Keywords:** gene expression profiles, pathways, human cancers, TCGA, intertumor homogeneity and heterogeneity

## Abstract

The homogeneity and heterogeneity in somatic mutations, copy number alterations and methylation across different cancer types have been extensively explored. However, the related exploration based on transcriptome data is lacking. In this study we explored gene expression profiles across 33 human cancer types using The Cancer Genome Atlas (TCGA) data. We identified consistently upregulated genes (such as E2F1, EZH2, FOXM1, MYBL2, PLK1, TTK, AURKA/B and BUB1) and consistently downregulated genes (such as SCARA5, MYOM1, NKAPL, PEG3, USP2, SLC5A7 and HMGCLL1) across various cancers. The dysregulation of these genes is likely to be associated with poor clinical outcomes in cancer. The dysregulated pathways commonly in cancers include cell cycle, DNA replication, repair, and recombination, Notch signaling, p53 signaling, Wnt signaling, TGFβ signaling, immune response etc. We also identified genes consistently upregulated or downregulated in highly-advanced cancers compared to lowly-advanced cancers. The highly (low) expressed genes in highly-advanced cancers are likely to have higher (lower) expression levels in cancers than in normal tissue, indicating that common gene expression perturbations drive cancer initiation and cancer progression. In addition, we identified a substantial number of genes exclusively dysregulated in a single cancer type or inconsistently dysregulated in different cancer types, demonstrating the intertumor heterogeneity. More importantly, we found a number of genes commonly dysregulated in various cancers such as PLP1, MYOM1, NKAPL and USP2 which were investigated in few cancer related studies, and thus represent our novel findings. Our study provides comprehensive portraits of transcriptional landscape of human cancers.

## INTRODUCTION

It has been recognized that cancer is associated with the genetic and genomic changes [1]. With the advance of microarray and next-generation sequencing technology, gene expression profiling has been widely used for identifying molecular biomarkers for cancer diagnosis, treatment and prognosis [2–6]. In addition, as the biology of cancer is extremely complicated, a simple genetic or genomic perspective is insufficient to understand it. The exploration of pathway perturbations in cancer is critical in comprehending the disease [7–9].

Cancers originating from different tissues or cell types vary in terms of their genomic profiles. Lawrence et al analyzed 27 cancer types and found that the median frequency of non-synonymous mutations varied by more than 1,000-fold across different cancer types [10]. The variation in mutation frequencies is mostly associated with cancer tissue type of origin where haematological and paediatric cancers have the lowest mutation frequencies while melanoma and lung cancers the highest mutation frequencies [10]. Zack et al analyzed the copy number profiles of 4,934 primary cancer specimens across 11 cancer types and found that the mean rate of somatic copy number alterations (SCNAs) varied across different cancer types with ovarian, cervix, breast and bladder cancers having a large number of SCNAs while leukemia and kidney cancers very few SCNAs [11]. Previous studies have also shown that numbers of methylomes and patterns of DNA methylation varied across different cancer types [12, 13].

Due to the varied genomic profiles, different cancer types may show different prognosis. Among all cancer types, pancreatic, lung, liver and esophageal cancers have the worst survival prognosis while prostate, thyroid and skin cancers have the best survival prognosis [14]. Moreover, different cancer types with the same genomic or genetic profiles may exhibit different responses to the same treatment strategies. For example, melanoma with the BRAF V600E mutation is highly responsive to the small-molecule inhibitor vemurafenib while colon cancers with the same mutation show a very limited response to this drug [15]. On the other hand, different cancer types show the major homogeneity. It has been appreciated that the multistep development of human tumors depends on the eight biological capabilities acquired [8, 16]. They include sustaining proliferative signaling, evading growth suppressors, resisting cell death, enabling replicative immortality, inducing angiogenesis, activating invasion and metastasis, reprogramming of energy metabolism and evading immune destruction [8, 16]. Although intertumor and intratumor heterogeneity extensively exists in genomic profiles [10, 17, 18], all the cancer driver genes are associated with 12 pathways that confer a selective growth advantage [9]. The 12 pathways include APC, Hedgehog, NOTCH, chromatin modification, transcriptional regulation, DNA damage control, TGF-β, MAPK, STAT, PI3K, RAS, cell cycle/apoptosis [9].

With the emergence of large-scale cancer genomics projects such as the International Cancer Genome Consortium (ICGC, http://icgc.org/) [19] and The Cancer Genome Atlas (TCGA, https://gdc-portal.nci.nih.gov/), the homogeneity and heterogeneity across different cancer types have been extensively explored [10–13, 17, 18, 20, 21]. The TCGA datasets cover 33 different cancer types and more than 10,000 cancer cases in total. Each TCGA cancer type contains different types of “omics” data, including: whole exome or genome sequencing; genomic DNA copy number arrays; DNA methylation; mRNA expression array and RNA-Seq data; microRNA sequencing; reverse-phase protein arrays; and clinical metadata. Based on the TCGA datasets, the tumor homogeneity and heterogeneity have been studied in various genomic profiles including somatic mutations [10, 18], SCNAs [11], and methylation [12, 13]. However, the exploration of homogeneity and heterogeneity across different cancer types specifically based on transcriptome data from large-scale cancer genomics projects such as TCGA is lacking.

In this study we explored gene expression profiles across 33 human cancer types in TCGA (Table 1). We identified dysregulated genes and pathways across different cancer types, and performed survival analyses based on expression profiles of the dysregulated genes. Tumors can be classified based on stage and grade. Tumor stage refers to the size and/or extent of the primary tumor and whether or not tumor cells have spread in the body [22]. Tumor grade refers to how abnormal the tumor cells and the tumor tissue look under a microscope compared to normal cells, indicative of how quickly a tumor is likely to grow and spread [22]. We identified differentially expressed (DE) genes and pathways between different stages and different grades of cancers, respectively. Furthermore, we explored the transcriptional homogeneity and heterogeneity across different cancer types. This study would bring additional insights into the biology of human cancers.

**Table 1:** 33 TCGA cancer types in which gene expression profiles were analyzed

Cancer^a^	Full name	# cancer samples	# normal samples	Stage^b^		Grade^c^	
				# early-stage	# late- stage	# low-grade	# high- grade
BLCA	bladder urothelial carcinoma	408	19	132	274	21	384
BRCA	breast invasive carcinoma	1100	112	801	270	NA	NA
CHOL	cholangiocarcinoma	36	9	28	8	16	20
COAD	colon adenocarcinoma	287	41	155	119	NA	NA
ESCA	esophageal carcinoma	185	11	97	65	95	49
GBM	glioblastoma multiforme	166	5	NA	NA	NA	NA
HNSC	head and neck squamous cell carcinoma	522	44	118	388	366	132
KICH	kidney chromophobe	66	25	46	20	NA	NA
KIRC	kidney renal clear cell carcinoma	534	72	324	207	243	282
KIRP	kidney renal papillary cell carcinoma	291	32	193	67	NA	NA
LIHC	liver hepatocellular carcinoma	373	50	257	90	232	134
LUAD	lung adenocarcinoma	517	59	397	110	NA	NA
LUSC	lung squamous cell carcinoma	501	51	406	91	NA	NA
PRAD	prostate adenocarcinoma	498	52	NA	NA	NA	NA
READ	rectum adenocarcinoma	95	10	38	46	NA	NA
STAD	stomach adenocarcinoma	415	35	180	212	160	246
THCA	thyroid carcinoma	509	59	334	165	NA	NA
UCEC	uterine corpus endometrial carcinoma	370	11	272	98	185	185
ACC	adrenocortical carcinoma	79	0	46	31	NA	NA
CESC	cervical squamous-cell carcinoma and endocervical adeno-carcinoma	306	3	231	66	153	119
DLBC	lymphoid neoplasm diffuse large B-cell lymphoma	48	0	25	17	NA	NA
LAML	acute myeloid leukemia	173	0	NA	NA	NA	NA
LGG	brain lower-grade glioma	530	0	NA	NA	249	265
OV	ovarian serous cystadeno-carcinoma	307	0	22	280	34	262
PAAD	pancreatic adeno-carcinoma	179	4	168	8	126	50
PCPG	pheochromocytoma and paraganglioma	184	3	NA	NA	NA	NA
SARC	sarcoma	263	2	NA	NA	NA	NA
SKCM	skincutaneous melanoma	472	1	217	193	NA	NA
TGCT	testicular germ-cell tumors	156	0	110	15	NA	NA
UCS	uterine carcino-sarcoma	57	0	27	30	NA	NA
UVM	uveal melanoma	80	0	36	44	NA	NA
MESO	mesothelioma	87	0	26	61	NA	NA
THYM	thymoma	120	2	97	21	NA	NA

^a^ 18 cancer types are underlined in which gene expression were compared between cancer and normal samples.

^b^ Numbers of early-stage (stage I-II) and late-stage (stage III-IV) cancer samples are shown.

^c^ Numbers of low-grade (G1-2) and high-grade (G3-4) cancer samples are shown.

* “NA” indicates no related information available.

## RESULTS

### Identification of DE genes between cancer and normal samples

**Figure 1:**
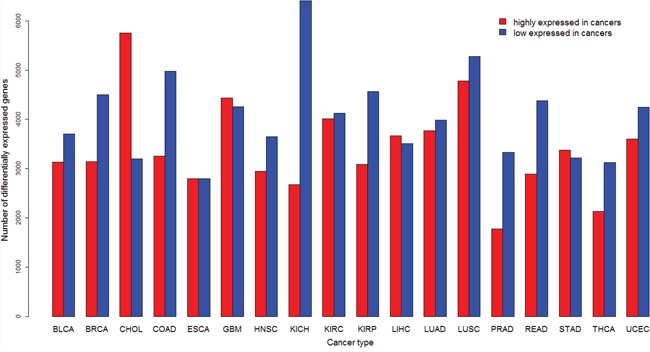
Number of differentially expressed (DE) genes between cancer and normal samples identified in each of the 18 cancer types

We compared gene expression levels between cancer and normal samples in 18 cancer types each of which contains at least five normal samples (Table 1). Supplementary Tables 1 and 2 list genes whose expression is significantly higher and lower in cancer than in normal samples (fold change > 1.5, false discovery rate (FDR) < 0.05), respectively. The numbers of DE genes vary across different cancer types (Figure 1). The most number (5,755) of genes are more highly expressed in CHOL, and the least number (1,780) in PRAD. The most number (6,404) of genes are more lowly expressed in KICH, and the least number (2,797) in ESCA. The median number of genes with higher and lower expression levels in cancers is 3,626 and is 4,055, respectively.

### Identification of genes consistently upregulated in different types of cancer

There are 51 genes consistently upregulated in all the 18 cancer types, and 52 genes consistently upregulated in 17 of the 18 cancer types compared to normal tissue (Supplementary Tables 3 and 4). Many of these genes have been reported to be overexpressed in cancers and associated with cancer progression and aggression. For example, E2F1 encodes a member of the E2F family of transcription factors (TFs) which play a crucial role in the control of cell cycle [23]. E2F1 has been shown to be upregulated in various cancers [24–26]. EZH2 encodes a member of the Polycomb-group (PcG) family that regulates cell generations. High EZH2 expression has been associated with different types of cancer [27]. Interestingly, the collaboration of EZH2 and E2F1 in transcriptional regulation has been observed in various cancers [28, 29]. The other TF genes such as FOXM1 [30–33], MYBL2 [34], NFE2L3 [35], and UHRF1 [36, 37] have been involved in various malignancies with overexpression. Among the 103 genes that are consistently upregulated in at least 17 of the 18 cancer types, there are 11 protein kinase encoding genes including PLK1, TTK, AURKA, AURKB, BUB1, BUB1B, GSG2, MELK, NEK2, PBK, and PKMYT1. Most of these kinase genes have been shown to be overexpressed in various cancers such as PLK1 [38–43], TTK [44–47], and AURKA/B [48–51]. These protein kinases are of particular interest because kinase inhibitors have been intensively investigated as a key class of anticancer drugs in clinical use or trials [52].

**Figure 2:**
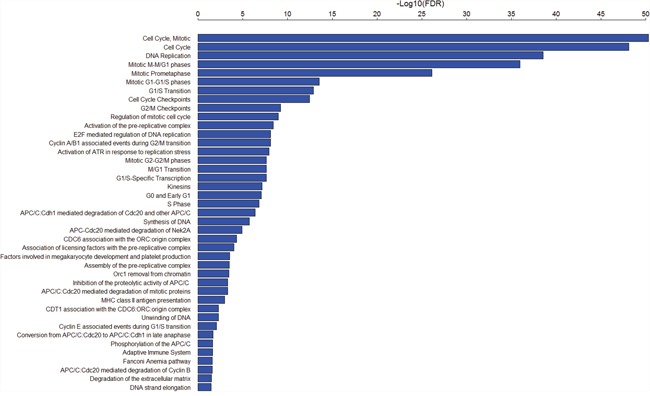
41 Rectome pathways significantly associated with the 103 genes upregulated in various cancers

**Figure 3:**
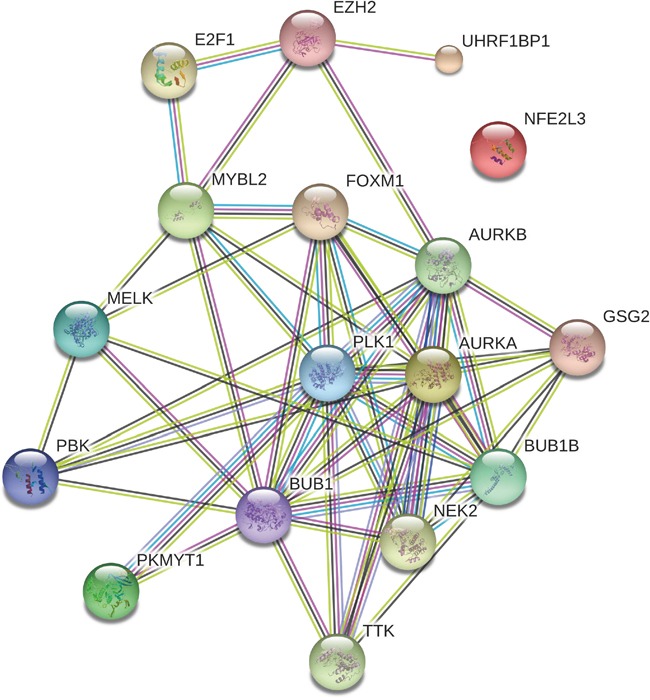
Interaction network associated with the seven transcriptional genes and the 11 protein kinase genes overexpressed in various cancers

Using Gene Set Enrichment Analysis (GSEA) software [53], we identified 41 Rectome pathways [54] significantly associated with the set of 103 genes (FDR<0.05, Figure 2 and Supplementary Table 5). Obviously, these gene products are significantly involved in cancer related pathways such as cell cycle, DNA replication and repair, and immune response. Network analysis of the gene set composed of the aforementioned seven TF genes (E2F1, EZH2, FOXM1, MYBL2, NFE2L3 and UHRF1) and the 11 protein kinase genes by STRING [55] shows that the protein products of these genes interact to each other (Figure 3). PLK1, a hub node in the network, interacts with 11 of the other 17 proteins (BUB1, BUB1B, PKMYT1, AURKA, AURKB, FOXM1, MYBL2, NEK2, TTK, PBK, and GSG2). As another hub node in the network, BUB1 interacts with 12 of the other 17 proteins (BUB1B, PLK1, MELK, MYBL2, PKMYT1, AURKA, AURKB, FOXM1, NEK2, TTK, PBK, and GSG2). The TF FOXM1 regulates seven protein kinases (BUB1, BUB1B, PLK1, MELK, AURKA, AURKB, and NEK2). The three TFs E2F1, EZH2 and MYBL2 interact with each other. This demonstrates that interactions of these oncoproteins may play an important role in the initiation and progression of various cancers.

**Figure 4:**
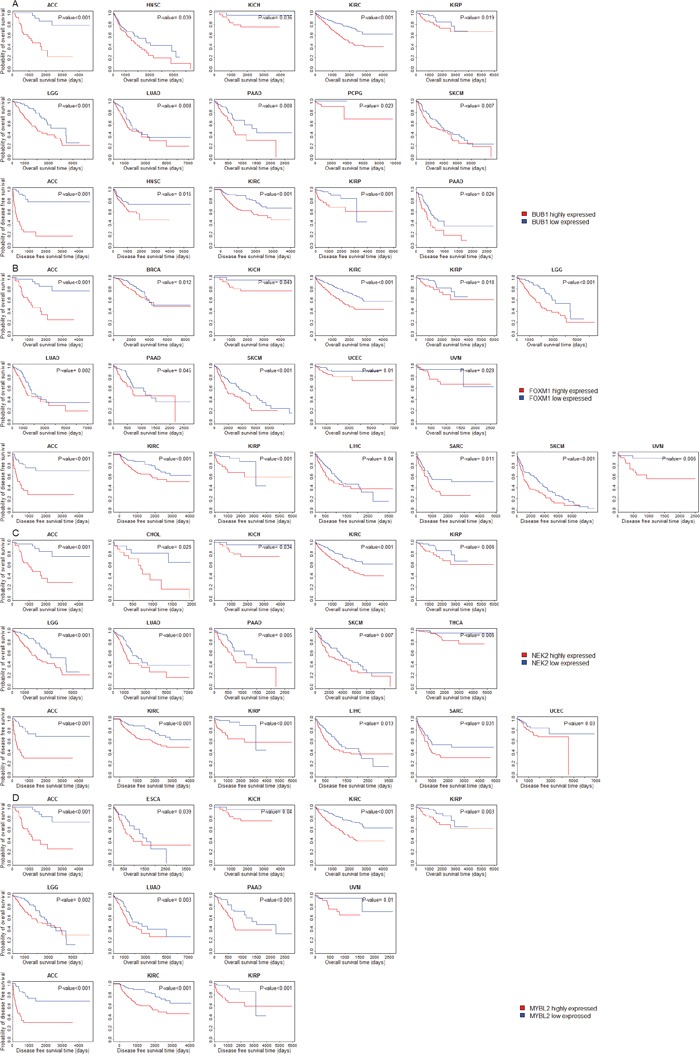
Survival analyses of cancer patients based on expression of the upregulated genes in cancers (log-rank test, unadjusted P-value < 0.05) **(A)** Compare survival time between BUB1 higher-expression-level and BUB1 lower-expression-level cancers; **(B)** Compare survival time between FOXM1 higher-expression-level and FOXM1 lower-expression-level cancers; **(C)** Compare survival time between NEK2 higher-expression-level and NEK2 lower-expression-level cancers; **(D)** Compare survival time between MYBL2 higher-expression-level and MYBL2 lower-expression-level cancers.

We compared overall survival (OS) and disease-free survival (DFS) between patients with higher expression levels and patients with lower expression levels of the 11 protein kinase genes and seven TF genes in 30 cancer types (three cancer types were excluded from the analysis due to lack of survival data). Kaplan-Meier survival curves show that higher expression levels of these genes are associated with significantly worse OS and DFS prognoses in various cancers (Figure 4 and Supplementary Figure 1). For example, patients with higher expression levels of BUB1 have worse OS prognoses than those with lower expression levels of BUB1 in 10 cancer types (ACC, HNSC, KICH, KIRC, KIRP, LGG, LUAD, PAAD, and SKCM), and worse DFS prognoses in nine cancer types (ACC, KIRC, KIRP, LGG, LIHC, LUAD, PAAD, SARC and UVM) (Figure 4A, log-rank test, unadjusted P-value < 0.05). Patients with higher expression levels of FOXM1 have worse OS prognoses than those with lower expression levels of FOXM1 in 11 cancer types (ACC, BRCA, KICH, KIRC, KIRP, LGG, LUAD, PAAD, SKCM, UCEC and UVM), and worse DFS prognoses in seven cancer types (ACC, KIRC, KIRP, LIHC, SARC, SKCM and UVM) (Figure 4B, log-rank test, unadjusted P-value < 0.05). Kaplan-Meier survival curves show that higher expression of NEK2 and MYBL2 is also associated with worse clinical outcomes in various cancers (Figure 4C, 4D). These results are consistent with previous studies showing that overexpression of BUB1, FOXM1, and NEK2 correlated with poor prognosis of cancers [56–58].

### Identification of genes consistently downregulated in different types of cancer

We identified 11 genes (SCARA5, PLP1, MYOM1, ADH1B, NKAPL, SYNE1, PEG3, USP2, PCDH9, SLC5A7 and HMGCLL1) which are consistently downregulated in all the 18 cancer types compared to normal tissue. Among the 11 genes, SCARA5 expression has been shown to be frequently downregulated in various cancers [59], and has been proposed as a novel tumor suppressor gene [60]. ADH1B was shown to be downregulated in colorectal cancer [61] and lung cancer [62]. SYNE1 has been shown to be downregulated in various human cancers [63]. PEG3 encodes a tumor suppressor and was downregulated in several cancer types [64–66]. The downregulation of PCDH9 has been shown to contribute to the development of various human cancers [67–69]. These previous studies confirm the results we obtained from the TCGA data analysis. However, few studies have shown that PLP1, MYOM1, NKAPL, and USP2 were consistently downregulated in various cancers. Thus, this analysis provides novel findings about these genes whose downregulation may play an important role in carcinogenesis. In addition, HMGCLL1 has been shown to be upregulated in several human cancers such as breast cancer [70] and brain cancer [71]. SLC5A7 had elevated mRNA expression in breast cancer cells compared with mammary epithelial cells [72]. These observations conflict with our results from the TCGA data analysis. Therefore, the roles of HMGCLL1 and SLC5A7 in carcinogenesis remain to be clarified.

**Figure 5:**
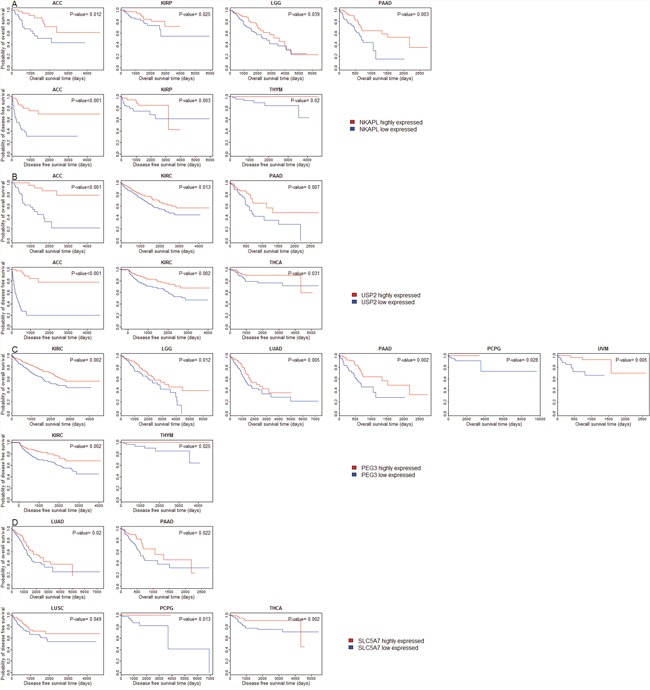
Survival analyses of cancer patients based on expression of the downregulated genes in cancers (log-rank test, unadjusted P-value < 0.05) **(A)** Compare survival time between NKAPL higher-expression-level and NKAPL lower-expression-level cancers; **(B)** Compare survival time between USP2 higher-expression-level and USP2 lower-expression-level cancers; **(C)** Compare survival time between PEG3 higher-expression-level and PEG3 lower-expression-level cancers; **(D)** Compare survival time between SLC5A7 higher-expression-level and SLC5A7 lower-expression-level cancers.

We compared OS and DFS between patients with higher expression levels and patients with lower expression levels of the 11 genes in the 30 cancer types. Kaplan-Meier survival curves show that higher expression levels of most of these genes are associated with better OS and DFS prognoses in multiple cancer types (Figure 5 and Supplementary Figure 1). For example, patients with higher expression levels of NKAPL have better OS prognoses than those with lower expression levels of NKAPL in four cancer types (ACC, KIRP, LGG, and PAAD), and better DFS prognoses in three cancer types (ACC, KIRP, and THYM) (Figure 5A, log-rank test, unadjusted P-value < 0.05). Patients with higher expression levels of USP2 have better OS prognoses than those with lower expression levels of USP2 in three cancer types (ACC, KIRC, and PAAD), and better DFS prognoses in three cancer types (ACC, KIRC, and THCA) (Figure 5B, log-rank test, unadjusted P-value < 0.05). Kaplan-Meier survival curves show that higher expression of PEG3 and SLC5A7 is also associated with better clinical outcomes in several cancer types (Figure 5C, 5D). Surprisingly, a literature survey shows that few studies have revealed the correlation between overexpression of these genes and beneficial clinical outcomes in cancers.

In addition, we identified 53 genes which are consistently downregulated in 17 of the 18 cancer types (Supplementary Tables 4 and 6). Pathway analysis did not find any significant pathway associated with the set of 64 genes consistently downregulated in at least 17 of the 18 cancer types (FDR<0.05).

### Identification of DE genes between highly-advanced and lowly-advanced cancers

**Figure 6:**
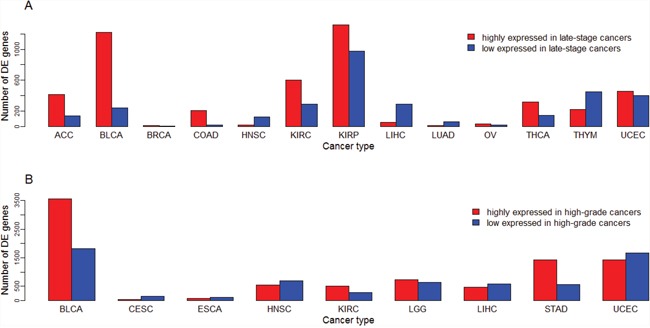
Number of differentially expressed (DE) genes between lowly-advanced and highly-advanced cancers across different cancer types **(A)** Number of DE genes between early-stage and late-stage cancers; **(B)** Number of DE genes between low-grade and high-grade cancers.

We compared gene expression levels between early-stage (stage I-II) and late-stage (stage III-IV) cancers, and between low-grade (G1-2) and high-grade (G3-4) cancers, respectively. We refer to early-stage or low-grade cancers as lowly-advanced cancers, and late-stage or high-grade cancers highly-advanced cancers. There are 27 and 12 cancer types whose clinical data contain stage and grade information, respectively (Table 1). Supplementary Table 7 presents the numbers of DE genes between highly-advanced and lowly-advanced cancers. Supplementary Tables 8 and 9 list genes whose expression is higher and lower in late-stage than in early-stage cancers, respectively. In 13 of the 27 cancer types there are DE genes between different stages of cancers (fold change > 1.5, FDR < 0.05). The numbers of DE genes between different stages of cancers vary across different cancer types (Figure 6A). In KIRP the most number (1,318) of genes are more highly expressed in late-stage than in early-stage cancers, and in the same cancer type the most number (975) of genes are more highly expressed in early-stage than in late-stage cancers. In contrast, in some other cancer types such as BRCA, only two and six genes were upregulated and downregulated in late-stage compared to early-stage cancers, respectively. In some cancer types such as KIRP, BLCA, KIRC, ACC, and COAD, the number of genes with higher expression levels in late-stage than in early-stage cancers are much higher than that of genes with lower expression levels in late-stage than in early-stage cancers. In some other cancer types such as THYM, LIHC, HNSC, and LUAD, the situation is just the opposite.

Supplementary Tables 10 and 11 list genes whose expression is higher and lower in high-grade than in low-grade cancers, respectively. In nine of the 12 cancer types there are DE genes between different grades of cancers (fold change > 1.5, FDR < 0.05). The numbers of DE genes between different grades of cancers also vary across different cancer types (Figure 6B). In BLCA the most number (3,572) of genes are more highly expressed in high-grade than in low-grade cancers, and in the same cancer type the most number (1,819) of genes are more highly expressed in low-grade than in high-grade cancers. In contrast, in ESCA only 79 and 118 genes were upregulated and downregulated in high-grade compared to low-grade cancers, respectively. In some cancer types such as STAD, the number of genes with higher expression levels in high-grade than in low-grade cancers are much higher than that of genes with lower expression levels in high-grade than in low-grade cancers (1,420 versus 573). In some other cancer types such as CESC, we observed the opposite situation (35 versus 156).

These results indicate that during cancer progression some cancers such as BLCA, KIRP, KIRC, HNSC, LIHC and UCEC exhibit expression disturbances in a large number of genes, while some other cancers such as BRCA, PAAD, LUAD, LUSC, and SKCM exhibit expression disturbances in a small number of genes. Interestingly, although both stage and grade indicate the degree of cancer progression, in some cancer types such as STAD, ESCA, and CESC, the markedly different numbers of DE genes between lowly-advanced and highly-advanced cancers were identified in the stage and grade phenotype comparisons (Figure 6A, 6B).

### Identification of genes upregulated in highly-advanced cancers

We identified 71 genes which are upregulated in late-stage compared to early-stage cancers in more than three cancer types (Supplementary Table 12). We call the 71 genes late-stage-activated (LSA) genes. Pathway analysis of the 71 LSA genes identified four significant Rectome pathways: extracellular matrix organization (P-value = 6.9*10^-15^), collagen formation (P-value = 2.1*10^-14^), cell surface interactions at the vascular wall (P-value = 9.4*10^-6^), and integrin cell surface interactions (P-value = 0.0002). Obviously, these pathways are associated with cancer progression and metastasis characteristics such as cell interaction, cell adhesion, and cell motility. Some of the LSA genes have been shown to be overexpressed in advanced cancers and be associated with unfavorable clinical outcomes such as SOX11 [73, 74], PTPRN [75], PNCK [76] and HMGA2 [77–79]. In addition, we identified 212 genes which are upregulated in high-grade compared to low-grade cancers in more than three cancer types (Supplementary Table 13). We call the 212 genes high-grade-activated (HGA) genes. Pathway analysis of these HGA genes identified 63 significant Rectome pathways (Supplementary Table 14). These pathways are mainly involved in cell cycle, DNA replication, and immune system whose dysregulations are the leading causes of cancer development [8, 9].

**Figure 7:**
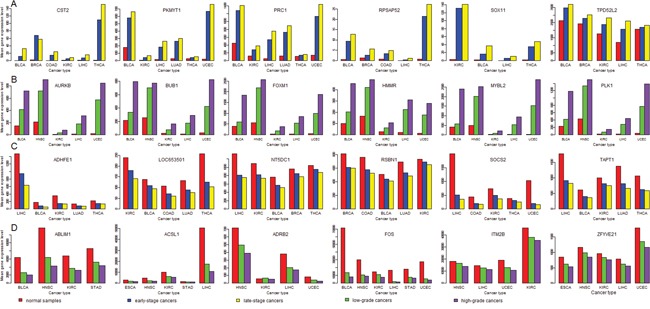
Genes have increased or decreased expression alterations from normal tissue to lowly-advanced cancers, and to highly-advanced cancers (Student's t test, P-value < 0.05) **(A)** Genes whose expression follows the pattern: late-stage cancers > early-stage cancers > normal tissue; **(B)** Genes whose expression follows the pattern: high-grade cancers > low-grade cancers > normal tissue; **(C)** Genes whose expression follows the pattern: late-stage cancers < early-stage cancers < normal tissue; **(D)** Genes whose expression follows the pattern: high-grade cancers < low-grade cancers < normal tissue.

There are much more HGA genes upregulated in cancers than HGA genes downregulated in cancers compared to normal tissue. For example, in more than nine (50%) of the 18 cancer types, 128 (60%) of the 212 HGA genes are upregulated in cancers, compared to 15 (7%) of the 212 HGA genes downregulated in cancers (Fisher's exact test, P-value < 2.2*10^-16^). Similarly, there are much more LSA genes upregulated in cancers than LSA genes downregulated in cancers compared to normal tissue. For example, in more than nine (50%) of the 18 cancer types, 26 (37%) of the 71 LSA genes are upregulated in cancers, compared to 9 (13%) of the 71 LSA genes downregulated in cancers (Fisher's exact test, P-value =0.0016). These results indicate that cancer initiation (normal tissue evolving into cancer tissue) and cancer progression (low-grade cancers evolving into high-grade cancers, or early-stage cancers evolving into late-stage cancers) may depend on many common changes in gene expression profiles. In fact, we found a number of genes whose expression follows this pattern: late-stage cancers > early-stage cancers > normal tissue. For example, in the eight cancer types (BLCA, BRCA, COAD, KIRC, LIHC, LUAD, THCA and UCEC) with both stage phenotype information and normal control samples, SOX11 expression follows the pattern in four cancer types (BLCA, KIRC, LIHC and THCA) (Figure 7A). Similarly, we found a number of genes whose expression follows this pattern: high-grade cancers > low-grade cancers > normal controls. For example, in the seven cancer types (BLCA, ESCA, HNSC, KIRC, LIHC, STAD and UCEC) with both grade phenotype information and normal control samples, expression of AURKB, BUB1, FOXM1, HMMR, MYBL2, and PLK1 follows the pattern in five cancer types (BLCA, HNSC, KIRC, LIHC, and UCEC) (Figure 7B). These results confirm that overexpression of PLK1 [38–42], BUB1 [80], AURKB [50], HMMR [81], FOXM1 [30–33], MYBL2 [34] and SOX11 [73, 74] are associated with both cancer onset and cancer progression.

Surprisingly, very few (only seven) genes are common between the LSA gene list and the HGA gene list. In fact, the gene expression profiling alteration (GEPA) from normal tissue to cancers is closer to the GEPA from low-grade to high-grade cancers than to that from early-stage to late-stage cancers. For example, 42 (82%) of the 51 genes overexpressed in all the 18 cancer types are included in the HGA gene list, compared to one (2%) of the 51 genes included in the LSA gene list (Fisher's exact test, P-value < 2.2*10^-16^). In more than nine (50%) of the 18 cancer types, 128 (60%) of the HGA genes are more highly expressed in cancers than in normal tissue, compared to 26 (37%) of the LSA genes (Fisher's exact test, P-value =0.0006). It is consistent with a recent study showing that cancer grade, but not stage, was driven by transcriptional alterations [82].

### Identification of genes downregulated in highly-advanced cancers

We identified 13 genes downregulated in late-stage compared to early-stage cancers in more than three cancer types (Supplementary Table 15). We call the 13 genes (DNASE1L3, CD1E, SLC44A4, PLIN5, IYD, RORC, GGT6, FBP1, ALDH1L1, PIGR, SPATA18, ARPP21 and CWH43) late-stage-inactivated (LSiA) genes. Pathway analysis of these LSiA genes did not find any significant Rectome pathway associated with them. In addition, we identified 58 genes downregulated in high-grade compared to low-grade cancers in more than three cancer types (Supplementary Table 16). We call the 58 genes high-grade-inactivated (HGiA) genes. Pathway analysis of these HGiA genes identified three significant Rectome pathways: biological oxidations, phase 1 - functionalization of compounds, and cytochrome P450 - arranged by substrate type. The associations between these pathways and cancer progression are unclear and remain to be investigated.

There are more HGiA genes downregulated in cancers than HGiA genes upregulated in cancers compared to normal tissue, although the difference is not significant. For example, in more than nine (50%) of the 18 cancer types, 9 (16%) of the 58 HGiA genes are downregulated in cancers, compared to 3 (5%) of the 58 HGiA genes upregulated in cancers (Fisher's exact test, P-value = 0.12). Similarly, there are more LSiA genes downregulated in cancers than LSiA genes upregulated in cancers, although the difference is not significant. Likewise, we found a number of genes whose expression follows this pattern: late-stage cancers < early-stage cancers < normal tissue (Figure 7C), such as ADHFE1, LOC653501, NT5DC1, RSBN1, SOCS2 and TAPT1 in five cancer types. We also found a number of genes whose expression follows this pattern: high-grade cancers < low-grade cancers < normal tissue (Figure 7D), such as ALDH1L1, WIF1, ACSL1, FOS, and ABLIM1 in at least four cancer types. ALDH1L1 [83, 84] and WIF1 [85, 86] have been shown to be ubiquitously downregulated in cancers, and their downregulation was associated with poor clinical outcomes in cancer.

Again, very few genes (only three genes ALDH1L1, SLC44A4 and IYD) are common between the LSiA gene list and the HGiA gene list. It strongly suggests that although both stage and grade reflect the status of cancer advancement in phenotype, they are markedly different in molecular levels.

### Pathway analyses of DE genes

**Figure 8:**
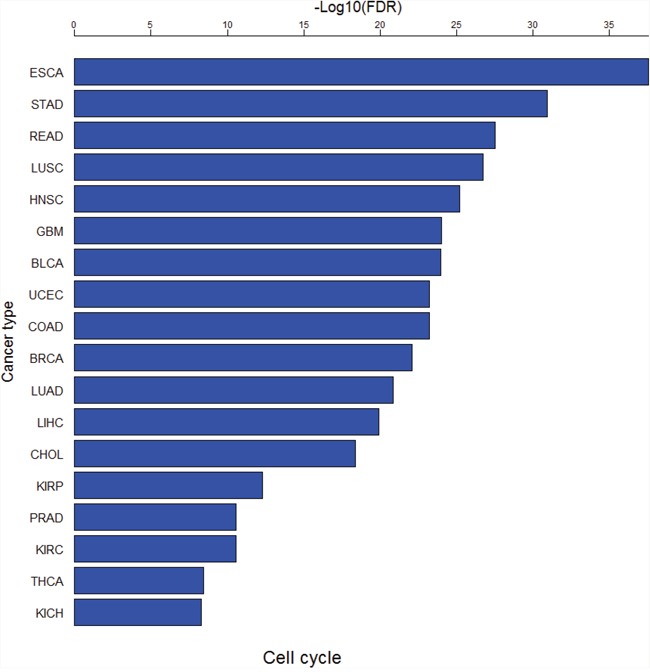
The cell cycle pathway is consistently upregulated in all the 18 cancer types

We used GSEA software [53] to perform pathway analyses of the DE genes between cancer and normal samples in each of the 18 cancer types. Supplementary Tables 17 and 18 show significant KEGG pathways [87] associated with the upregulated and downregulated genes in cancers (FDR<0.05), respectively. There are 22 pathways only associated with upregulated genes, and six pathways only associated with downregulated genes in at least one cancer type (Supplementary Table 19). Remarkably, the cell cycle pathway is consistently upregulated in all the 18 cancer types (Figure 8), suggesting that hyperactivation of this pathway is a common mechanism underlying cancer initiation and progression. The other dysregulated pathways in cancer such as DNA replication, repair, and recombination, and Notch signaling [9] were also identified to be aberrantly activated in various cancers in this study (Supplementary Table 19).

Pathway analyses of the DE genes between early-stage and late-stage cancers in each of the 13 cancer types identified 28 and 48 significant KEGG pathways associated only with the upregulated genes and only with the downregulated genes in late-stage cancers in at least one cancer type, respectively (FDR<0.05, Supplementary Table 20). Pathway analyses of the DE genes between low-grade and high-grade cancers in each of the nine cancer types identified 44 and 29 significant KEGG pathways associated only with the upregulated genes and only with the downregulated genes in high-grade cancers in at least one cancer type, respectively (FDR<0.05, Supplementary Table 21). Supplementary Tables 20 and 21 show that a number of pathways are upregulated in highly-advanced cancers such as cell cycle, ECM receptor interaction, DNA replication, DNA mismatch repair, homologous recombination, antigen processing and presentation, and nicotinate and nicotinamide metabolism. Among them, the pathways cell cycle, DNA replication, DNA mismatch repair and homologous recombination have also been identified to be consistently upregulated in various cancers (Supplementary Table 19). It suggests that hyperactivation of these pathways drives both cancer onset and cancer progression. The pathways significantly downregulated in highly-advanced cancers are mainly involved in metabolism regulation such as ether lipid metabolism, alpha linolenic acid metabolism, glycolysis gluconeogenesis, histidine metabolism, butanoate metabolism, beta alanine metabolism, propanoate metabolism, pyruvate metabolism, and phenylalanine metabolism.

### Intertumor homogeneity and heterogeneity in gene expression profiles

We identified a number of genes which are consistently upregulated or downregulated in various cancers. For example, there are 51 genes consistently upregulated and 11 genes consistently downregulated in all the 18 cancer types (Supplementary Tables 22, 23). Moreover, the cell cycle pathway is consistently upregulated in all the 18 cancer types (Supplementary Table 19). When comparing highly-advanced with lowly-advanced cancers, we also identified a number of genes consistently upregulated or downregulated in highly-advanced cancers (Supplementary Tables 24, 25, 26, 27). For example, there are 70 and 12 genes consistently upregulated and consistently downregulated in late-stage cancers in at least four cancer types, respectively. There are 49 and six genes consistently upregulated and consistently downregulated in high-grade cancers in at least five cancer types, respectively. These results demonstrate that there exist common genes and pathways whose dysregulations lead to the development of different types of cancer.

**Figure 9:**
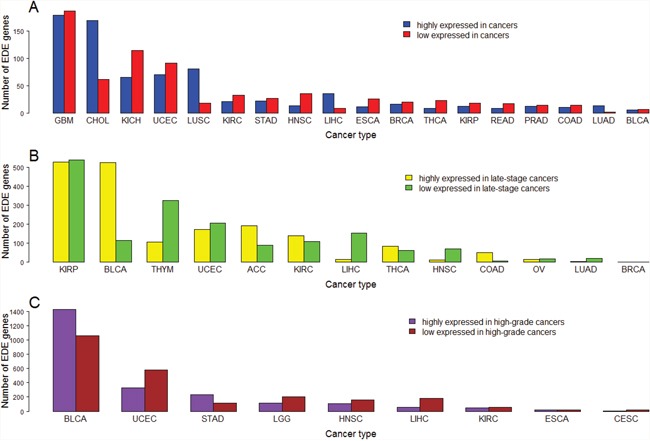
Number of exclusively differentially expressed (EDE) genes identified in a single cancer type **(A)** Number of EDE genes between cancer and normal samples; **(B)** Number of EDE genes between early-stage and late-stage cancers; **(C)** Number of EDE genes between low-grade and high-grade cancers.

In addition, we identified a number of genes which are upregulated in some cancer types while downregulated in other cancer types (Supplementary Table 28). For example, there are 171 genes which are upregulated in at least six cancer types while downregulated in other at least six cancer types, respectively. We also identified a number of genes which are upregulated in highly-advanced cancers in some cancer types while downregulated in other cancer types (Supplementary Tables 29, 30). For example, there are 15 genes which are upregulated in late-stage cancers in at least two cancer types while downregulated in other at least two cancer types (Supplementary Table 29), and 110 genes which are upregulated in high-grade cancers in at least two cancer types while downregulated in other at least two cancer types (Supplementary Table 30). Moreover, we identified a number of genes exclusively dysregulated in a single cancer type (Supplementary Table 31, Figure 9). For example, there are 178 and 186 genes upregulated and downregulated in GBM, respectively, but not in the other 17 cancer types. The number of exclusively differentially expressed (EDE) genes in GBM is the most among the 18 cancer types, suggesting the major specificity in transcriptional dysregulations that underly the development of GBM. In fact, a recent study has shown that GBM is different from other cancers in that TP53-mutated GBM has a better prognosis than TP53-wildtype GBM while most of other cancers have worse prognoses when TP53 mutated [88]. Interestingly, although few genes are exclusively differentially expressed between BLCA and normal tissue, a large number of genes are exclusively differentially expressed between highly-advanced and lowly-advanced BLCA. It may suggest that the BLCA progression not BLCA onset is associated with substantial specific gene expression disturbances.

## DISCUSSION

In this study we performed extensive analyses of gene expression and clinical data from 33 TCGA cancer type-specific datasets. We identified upregulated and downregulated genes and pathways commonly across different cancer types. Many TF genes (such as E2F1, EZH2, FOXM1 and MYBL2) and protein kinase genes (such as PLK1, TTK, AURKA, AURKB, BUB1, MELK, NEK2, PBK and PKMYT1) are overexpressed in various cancers, and their overexpression is associated with poor clinical outcomes in cancer (Supplementary Figure 1). Clearly, these genes are oncogenes whose hyperactivation leads to cancer initiation and progression. In contrast, lower expression of the downregulated genes (such as SCARA5, MYOM1, NKAPL, PEG3, USP2, SLC5A7 and HMGCLL1) in various cancers is associated with poor clinical outcomes in cancer (Supplementary Figure 1). These genes are tumor suppressor genes whose hypoactivation leads to cancer initiation and progression. Although many of the identified genes such as E2F1, EZH2, FOXM1, PLK1, TTK, AURKA, AURKB, and BUB1 have been revealed to be dysregulated in various cancers by previous studies, many other genes such as PLP1, MYOM1, NKAPL, and USP2 were investigated in few cancer-related studies, and thus represent our novel findings. Pathway analyses show that the cell cycle pathway is upregulated commonly in all the cancer types. The other dysregulated pathways in various cancers include DNA replication, repair, and recombination, Notch signaling etc. Moreover, we identified a number of genes consistently upregulated or downregulated in highly-advanced relative to lowly-advanced cancers. An interesting finding is that those genes with higher (lower) expression levels in highly-advanced than in lowly-advanced cancers are likely to have higher (lower) expression levels in cancers than in normal tissue. It suggests that common molecular perturbations drive cancer evolution from normal tissue to early cancer, and from early cancer to late cancer. However, we also found many genes which are upregulated in some cancer types while downregulated in other cancer types, and many genes which are exclusively dysregulated in a single cancer type. It suggests that there exists extensive intertumor heterogeneity in genomic profiles.

The commonly dysregulated genes and pathways identified in various cancers may involve attractive therapeutic targets for cancer. For example, since the cell cycle pathway is consistently hyperactivated in cancers, development of cell-cycle inhibitors may be effective in treatment of a wide type of cancers. We have identified a number of protein kinase genes which are upregulated commonly in cancers and are involved in the cell cycle regulation such as PLK1, TTK, BUB1, BUB1B, and PKMYT1. Development of small molecule inhibitors targeting these protein kinases could be a promising direction for curing cancer. On the other hand, the identification of a number of genes exclusively dysregulated in a single cancer type indicates that an individual cancer type may need its own specific therapeutic strategies in addition to the common strategies in cancer therapy. Furthermore, the identification of a considerable number of genes commonly dysregulated across various cancers while with different directions indicates the complexities of cancer therapy unless the dysregulation of these genes is a passenger event.

A limitation of the present study is that a small number of normal samples in some cancer types such as GBM and CHOL could compromise the validity of the results from the analyses of DE genes between normal and cancer samples. To overcome the limitation, the method of merging samples based on the body sites of cancer initiation (such as brain cancer, lung cancer, gastrointestinal cancer, kidney cancer, blood cancer, etc.) can be used. This is a direction for our future studies.

In addition, many upstream factors may affect expression of mRNAs (genes) in cancers such as gene mutations, DNA copy number alterations, DNA methylation, microRNA expression, and expression change of regulators. Combined analysis of other genomic profiles with gene expression profiles may gain more in-depth insights into the mechanism underlying oncogenesis [89]. Besides, the study of downstream products (proteins) of genes is crucial in cancer research since proteins directly determine cell function and fate [90]. Integration of different “omics” data to explore oncogenesis in a wide type of cancers represents a promising direction for cancer research.

## MATERIALS AND METHODS

### Materials

We downloaded RNA-Seq gene expression data (Level 3), and clinical data for all of the 33 cancer types for which data are available from the TCGA data portal (https://gdc-portal.nci.nih.gov/). For survival analyses we used clinical data from FireBrowse (http://gdac.broadinstitute.org/).

### Class comparison to identify differentially-expressed genes

We first normalized the gene expression data by base-2 log transformation, and then identified DE genes between two classes of samples using Student's *t* test. We used FDR to adjust for multiple tests. The FDR was estimated using the Benjami and Hochberg (BH) method [91]. We used the threshold of FDR < 0.05 and mean gene-expression fold-change > 1.5 to identify the DE genes.

### Gene-set enrichment analysis

We performed pathway analysis of gene sets using the GSEA tool [53], and network analysis of gene sets by STRING [55].

### Survival analyses

We performed survival analyses of TCGA patients based on gene expression data. Kaplan-Meier survival curves were used to show the survival (OS or DFS) differences between gene higher-expression-level patients and lower-expression-level patients. Gene higher-expression-level and lower-expression-level patients were determined by the median values of gene expression levels. If the gene expression level in a patient was higher than the median value, the patient was classified as gene higher-expression-level; otherwise as gene lower-expression-level. We used the log-rank test to calculate the significance of survival-time differences between two classes of patients with a threshold of P-value < 0.05.

## CONCLUSION

The present study provides comprehensive portraits of transcriptional landscape of human cancers, showing extensive intertumor homogeneity and heterogeneity in genomic profiles. This work would bring new insights into the biology of human cancers.

## SUPPLEMENTARY MATERIALS FIGURES AND TABLES


































