# Supplementary material for: Transcriptional landscape of human cancers
Source: Oncotarget. 2017 Mar 2;8(21):34534–51. doi: 10.18632/oncotarget.15837 (PMC5470989; doi:10.18632/oncotarget.15837)
Supplement: Supplementary file 1 [file oncotarget-08-34534-s001.pdf]

Transcriptional landscape of human cancers

SUPPLEMENTARY FIGURE AND TABLES

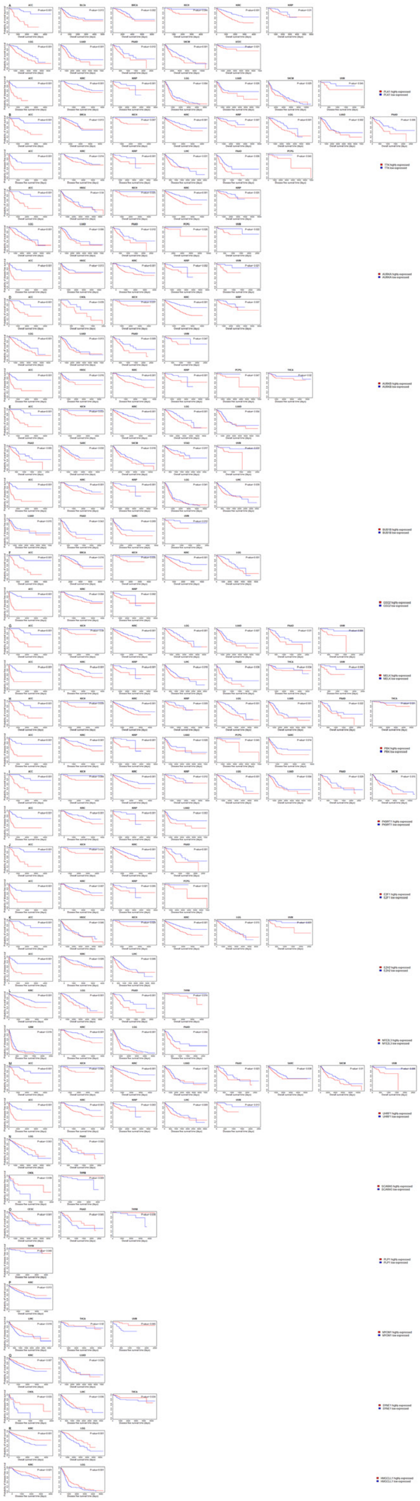

Supplementary Figure 1: Survival analyses of cancer patients based on expression of the dysregulated genes in cancers (log-rank test, unadjusted P-value < 0.05).

Supplementary Table 1: Genes whose expression is significantly higher in cancer than in normal tissue

See Supplementary File 1

Supplementary Table 2: Genes whose expression is significantly lower in cancer than in normal tissue

See Supplementary File 2

Supplementary Table 3: Genes upregulated in various cancer types

See Supplementary File 3

Supplementary Table 4: Genes dysregulated in almost all cancer types

See Supplementary File 3

Supplementary Table 5: Pathways significantly associated with the set of 103 genes overexpressed in at least 17 of the 18 cancer types

See Supplementary File 3

Supplementary Table 6: Genes downregulated in various cancer types

See Supplementary File 3

Supplementary Table 7: Comparison of gene expression between lowly-advanced and highly-advanced cancers

See Supplementary File 4

Supplementary Table 8: Genes whose expression is higher in late-stage cancers than in early-stage cancers

See Supplementary File 5

Supplementary Table 9: Genes whose expression is lower in late-stage cancers than in early-stage cancers

See Supplementary File 6

Supplementary Table 10: Genes whose expression is higher in high-grade cancers than in low-grade cancers

See Supplementary File 7

Supplementary Table 11: Genes whose expression is lower in high-grade cancers than in low-grade cancers

See Supplementary File 8

Supplementary Table 12: Genes upregulated in late-stage cancers compared to early-stage cancers

See Supplementary File 9

Supplementary Table 13: Genes upregulated in high-grade cancers compared to low-grade cancers

See Supplementary File 9

Supplementary Table 14: Pathways significantly associated with the 212 high-grade-activated (HGA) genes

See Supplementary File 9

Supplementary Table 15: Genes downregulated in late-stage cancers compared to early-stage cancers

See Supplementary File 9

Supplementary Table 16: Genes downregulated in high-grade cancers compared to low-grade cancers

See Supplementary File 9

Supplementary Table 17: Pathways significantly associated with the upregulated genes in cancers

See Supplementary File 10

Supplementary Table 18: Pathways significantly associated with the downregulated genes in cancers

See Supplementary File 11

Supplementary Table 19: Pathways significantly associated with only the upregulated genes and the downregulated genes in cancers, respectively

See Supplementary File 12

Supplementary Table 20: Pathways significantly associated with only the upregulated and downregulated genes in late-stage cancers compared to early-stage cancers, respectively

See Supplementary File 12

Supplementary Table 21: Pathways significantly associated with only the upregulated and downregulated genes in high-grade cancers compared to low-grade cancers, respectively

See Supplementary File 12

Supplementary Table 22: Genes only upregulated in cancers compared to normal tissue

See Supplementary File 13

Supplementary Table 23: Genes only downregulated in cancers compared to normal tissue

See Supplementary File 13

Supplementary Table 24: Genes only upregulated in late-stage cancers compared to early-stage cancers

See Supplementary File 14

Supplementary Table 25: Genes only downregulated in late-stage cancers compared to early-stage cancers

See Supplementary File 14

Supplementary Table 26: Genes only upregulated in high-grade cancers compared to low-grade cancers

See Supplementary File 14

Supplementary Table 27: Genes only downregulated in high-grade cancers compared to low-grade cancers

See Supplementary File 14

Supplementary Table 28: Genes upregulated in some cancers, and downregulated in some other cancers compared to normal tissue

See Supplementary File 15

Supplementary Table 29: Genes upregulated in late-stage cancers in some cancer types, and downregulated in late-stage cancers in some other cancer types compared to early-stage cancers

See Supplementary File 15

Supplementary Table 30: Genes upregulated in high-grade cancers in some cancer types, and downregulated in high-grade cancers in some other cancer types compared to low-grade cancers

See Supplementary File 15

Supplementary Table 31: Genes exclusively dysregulated in a single cancer type

See Supplementary File 16
